# Influence of physical activity on problematic smartphone use in medical students: mediating effects of social anxiety and the moderating role of gender

**DOI:** 10.3389/fpsyg.2024.1445847

**Published:** 2024-08-29

**Authors:** Yanhong Song, Guofeng Zhang, Ningning Liu, Yaqi Zhang, Jinghua Zhai, Xingmeng Niu, Yan Liu

**Affiliations:** ^1^College of Continuing Education, Jining Medical University, Jining, China; ^2^Basic Department, Jining Polytechnic, Jining, China; ^3^College of Basic Medical Sciences, Jining Medical University, Jining, China; ^4^Henan Key Laboratory of Medical Tissue Regeneration, Xinxiang Medical University, Xinxiang, China; ^5^College of Public Health, Jining Medical University, Jining, China; ^6^School of Public Health, Shandong Second University, Weifang, China

**Keywords:** physical activity, problematic smartphone use, college students, social anxiety, gender

## Abstract

**Objective:**

This study investigates the mediating role of social anxiety in the relationship between physical activity and problematic smartphone use among college students, and examines the moderating role of gender within this model.

**Methods:**

From April to May 2023, a survey was conducted involving 2905 undergraduate students from various medical schools in Shandong, China. Participants completed the Physical Activity Rating Scale (PARS-3), Mobile Phone Addiction Tendency Scale for College Students (MPATS), and the Social Anxiety Scale (IAS). Descriptive statistics, correlation analysis, and hierarchical regression analysis and other methods were employed to explore the relationships between the variables. The mediating effect of social anxiety on physical activity and mobile phone addiction was assessed using the bootstrapping method, while the moderating role of gender on social anxiety and mobile phone addiction was evaluated using the PROCESS macro's model 14.

**Results:**

The findings revealed that the scores of problematic smartphone use among medical students was 44.00 (35.00, 50.00), physical activity score was 16.00 (8.00, 32.00), and social anxiety score was 45.00 (42.00, 52.00). Physical activity was significantly and negatively correlated with both problematic smartphone use and social anxiety (*P* < 0.001), and social anxiety was significantly and positively correlated with problematic smartphone use (*P* < 0.001). Social anxiety partially mediated the effect between physical activity and problematic smartphone use (β = −0.04, 95%*CI* = −0.05 to −0.02), with a mediation effect proportion of 57.14%. Sex played a moderating role between social anxiety and problematic smartphone use (β = −0.30, 95%*CI* = −0.39 to −0.21).

**Conclusion:**

Physical activity influences problematic smartphone use among medical students both directly and indirectly through social anxiety. Sex significantly moderates the influence of social anxiety on problematic smartphone use, highlighting the need for gender-specific interventions in this demographic.

## Introduction

With the iteration of mobile terminal technology and the development of Internet smart devices, the global usage of mobile terminals, particularly smartphones, is growing steadily (Bin and Xiaoyong, 2017). In August 2023, the China Internet Network Information Center (CNNIC) released the 52nd Statistical report on the Development of the Internet in China, revealing that the Internet penetration rate in China has reached 76.4% (China Internet Network Information Center, 2023). Among them, smartphones have become the primary devices for network access due to their convenience and complete functions. Research on the theory of network use and satisfaction point out that the frequent use of network devices such as mobile phones can meet the psychological needs of college students in terms of social interaction and entertainment, thus leading to the problematic smartphone use (Liu et al., 2016).

Mobile phone addiction, recognized as a behavioral addiction (Beranuy et al., 2009), poses significant risks to physical and mental health, especially among college students facing high stress and academic pressures. This addiction often serves as a coping mechanism for stress and negative emotions (Garland and Howard, 2018; Mehmood et al., 2021), with research indicating a higher prevalence among medical students-−41.93% compared to 36.6% in the general college population (Zhong et al., 2022; Lai et al., 2023). The urgent need to address this issue highlights the importance of developing effective interventions to enhance mental health and curb this dependency.

Physical exercise emerges as a potent mitigator of problematic smartphone use (Yang et al., 2021). Activities such as running, swimming, and fitness training not only promote healthy behaviors but also fulfill unmet psychological needs, reducing the likelihood of addiction (Yang et al., 2021; Cai et al., 2023; Su et al., 2024). The release of dopamine during physical activity enhances self-efficacy and influences addictive behaviors (Ren and Li, 2020; Lei and Russell, 2021), suggesting that regular exercise could effectively combat smartphone addiction.

Moreover, social anxiety exacerbates problematic smartphone use (Gong et al., 2023a). This emotional disorder, characterized by a fear of negative evaluation in social situations, drives individuals—particularly females, who often place a higher value on interpersonal connections—to rely on smartphones for social interaction (Lee-Won and Herzog, 2015). Uses and Gratifications Theory suggests that individuals satisfy specific inner needs through certain medium (excessive use of mobile phones). As a tool with social interaction nature, people with social anxiety will rely more on mobile phones for interpersonal interactions, thus are more likely to develop mobile phone addiction (Busch, 2021). Engaging in group sports can alleviate social anxiety by improving mood, reducing negative emotions, and building social skills and relationships (Ren and Li, 2020), thus addressing both the physical and psychological aspects of smartphone addiction (Li and Jia, 2015).

Previous studies showed that physical activity is negatively correlated with both problematic smartphone use and social anxiety (Chen et al., 2016). Systematic physical exercise has been demonstrated to effectively alleviate the tendency of problematic smartphone use, aligning with the results of previous studies (Xiang et al., 2019). Additionally, social anxiety is positively correlated with problematic smartphone use (Li et al., 2022; Tong and Meng, 2023). Studies also suggest that college students' physical exercise negatively predicts problematic smartphone use, and that participating in group sports activities helps obtain social support, promote the development of social ability, establish good interpersonal relationships, and effectively alleviate social anxiety (Liu et al., 2022; Gong et al., 2023b). However, there is a notable gap in research exploring the mediating role of social anxiety between physical activity and problematic smartphone use, particularly among medical students. Given their critical societal role, emphasizing “life and health,” it is imperative to address the excessive reliance of medical students on mobile phones and enhance their mental health (Grover et al., 2018).

This study aims to develop a mediating model to investigate the relationship among physical activity, social anxiety and problematic smartphone use, proposing the following hypotheses: (1) Physical activity would be inversely related to problematic smartphone use among medical students; (2) Social anxiety would mediate the relationship between physical activity and problematic smartphone use of medical students; (3) Gender would moderate the interaction between social anxiety and problematic smartphone use.

## Methods and materials

### Participants and data collection

From April to May in 2023, a total of 3216 undergraduates from several medical colleges in Shandong Province, China, were selected by simple and convenient sampling. To ensure the quality of the survey, two psychology students, who had received professional training were selected as the chief examiners of this survey. Participation was voluntary, and students completed the questions anonymously through the online questionnaire tool (https://www.wjx.cn/), and the answering time was about 20 min. Out of the 3,216 questionnaires distributed, 311 were discarded due to incomplete answers or duplications, yielding 2,905 valid questionnaires were finally recovered, with an effective response rate of 90.3%. The study subjects were aged between 18 and 22 years [(19.31 ± 0.95) years]. Among them, 1,181 (40.65%) were and 1,724 (59.35%) were female. Each participant gave written informed consent, and all protocols were approved by the Science Ethics Committee of Jining Medical College.

The study utilized the Questionnaire Star Platform (https://www.wjx.cn/) to conduct the survey, which prevents multiple submissions from the same IP address to ensure data integrity. Before the questionnaire was issued, the subjects used unified instruction to explain the purpose, method and significance of the research. Throughout the survey, standardized guidance was provided to facilitate the completion of the questionnaires and enhance response quality. Upon collection, the responses were promptly checked to ensure the integrity and standardization of the questionnaires, discarding any non-standard questionnaires to retain the valid questionnaires.

### Measures

#### Demographic information

A self-designed questionnaire was used to investigate the general demographic data of medical students, including gender, age, etc. We include 3,216 undergraduates. Of these, 311 withdrew or were withdrawn. Reasons for withdrawal included invalid questionnaires and not meeting study requirements. Their average age was 19.31 ± 0.95 years old. Among them, 1,181 (40.65%) were and 1,724 (59.35%) were female. There were no significant differences in or other demographic or clinical characteristics.

#### Physical activity rating scale (PARS-3)

The PARS-3 scale (Fu et al., 2023) compiled by Japanese scholar Kimio Hashimoto and revised by domestic scholar Liang et al. was used to measure the amount of physical activity of medical students. The scale contains 4 questions and 3 dimensions, namely, exercise intensity, exercise time and exercise frequency. Likert5-point scoring method is adopted for each question. Exercise intensity and frequency are divided into 1 and 5 points respectively, and exercise time is divided into 0 and 4 points respectively. The total score is the product of exercise intensity, exercise time and exercise frequency. The higher the score, the greater the amount of exercise: < 19 points = small amount of exercise, 20~42 points = medium amount of exercise, and more than 42 points = large amount of exercise. The test-retest reliability of the scale is 0.82 (Fu et al., 2023).

#### Mobile phone addiction tendency scale for college students

The MPATS scale compiled by Xiong et al. (2012) was used to measure the problematic smartphone use tendency of college students, which includes 16 questions and 4 dimensions, namely withdrawal symptoms, prominent behavior, social comfort and mood change. Each question is scored on a scale of 1 to 5, with the total score ranging from 16 to 80, and higher score indicating the higher tendency to become addicted to mobile phones. The Cronbach's α coefficient of this scale in this study was 0.928.

#### Interaction anxiousness scale (IAS)

The IAS scale revised by Peng (2004) was used to measure the severity of social anxiety of medical students. This scale includes 15 questions, each of which was scored using Likert 5-point scoring method, with the lowest score being 1 point (completely inconsistent) and the highest score being 5 points (completely consistent), with the total score ranging from 15 to 75 points. The higher the score, the higher the severity of individual's social anxiety. The Cronbach's α coefficient of the scale in this study was 0.852.

### Statistical analysis

All data were analyzed by SPSS 26.0 software. The quantitative data (scores of physical activity, problematic smartphone use and social anxiety) presented non-normal distribution, represented by M (P25, P75). Mann-Whitney U was used to test the gender differences, and Spear-man method was used to analyze the correlation between variables. The PROCESS macro program of SPSS 26.0 was used to analyze the mediating role of social anxiety in physical activity and problematic smartphone use and the moderating role of gender. The 95% confidence interval of the effect value was calculated through Bootstrap method. If 0 was not included, the mediating effect was significant.

## Results

### Differences in scores of physical activity, social anxiety and problematic smartphone use among medical students of different genders

The study included a total of 2,905 undergraduate students from different medical colleges in Shandong Province, China. The participants were recruited through convenient sampling. The study was conducted in April and May 2023. The participants' ages ranged from 18 to 22 years, with a mean age of 19.31 years (SD = 0.95). Among the participants, 1,181 (40.65%) were male and 1,724 (59.35%) were female.

The scores of medical students' physical activity, social anxiety and problematic smartphone use were 16.00 (8.00, 32.00), 44.00 (35.00, 50.00), and 45.00 (42.00, 52.00) respectively. Mann-Whitney U test results showed that there were significant gender differences in the scores of physical activity (Z = −12.179, *P* < 0.001), social anxiety (Z = −8.413, *P* < 0.001), and problematic smartphone use (Z = −4.515, *P* < 0.001) (Z physical activity = −12.179, *P* < 0.001; Z social anxiety = −8.413, *P* < 0.001; Z problematic smartphone use = −4.515, *P* < 0.001), the specific results are shown in Table 1.

**Table 1:** The difference analysis of physical activity, social anxiety and mobile phone ad-diction among medical students of different genders.

**Variate**	**Total (*n =* 2,905)**	**Male (*n =* 1,181)**	**Female (*n =* 1,724)**	***Z***	***P***
Physical activity	16.00 (8.00, 32.00)	24.00 (12.00, 40.00)	16.00 (8.00, 24.00)	−12.179	< 0.001
Social anxiety	45.00 (42.00, 52.00)	45.00 (39.00, 49.00)	46.00 (43.00, 54.00)	−8.413	< 0.001
Problematic smartphone use	44.00 (35.00, 50.00)	43.00 (33.00, 48.00)	44.00 (36.00, 50.00)	−4.515	< 0.001

### Correlation analysis of physical activity, social anxiety and problematic smartphone use among medical students

Spearman correlation analysis was carried out among physical activity, social anxiety and problematic smartphone use. The results showed that physical activity was significantly negatively correlated with problematic smartphone use and social anxiety (r = −0.131, *P* < 0.001; r = −0.131, *P* < 0.001), while social anxiety was positively correlated with problematic smartphone use (r = 0.483, *P* < 0.001). The results are shown in Table 2.

**Table 2:** Correlation analysis of physical activity, social anxiety and problematic smartphone use.

**Variate**	**Physical activity**	**Social anxiety**	**Problematic smartphone use**
Physical activity	1		
Social anxiety	−0.131^a^	1	
Problematic smartphone use	−0.131^a^	0.483^a^	1

^a^P < 0.001.

### Analysis of the mediating effect of social anxiety between physical activity and problematic smartphone use of medical students

**Figure 1:**
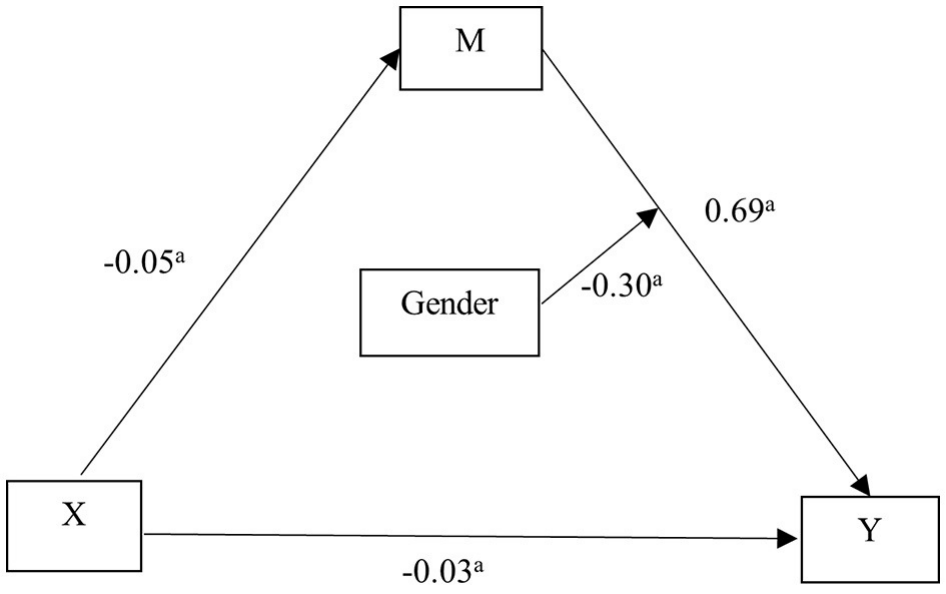
The mediating effect model of social anxiety between physical activity and problematic smartphone use. ^a^*P* < 0.001. Independent variable (X): Physical activity; Dependent variable (Y): Problematic smartphone use; Mediating variable (M): Social anxiety; Control variables: Age and gender. Physical activity negatively predicted problematic smartphone use. Even after accounting for social anxiety, the negative predictive effect of physical activity on problematic smartphone use remains significant (β = −0.03, *P* = 0.002). Conversely, social anxiety positively predicted problematic smartphone use (β = 0.69, *P* < 0.001).

Taking physical activity as the independent variable (X), problematic smartphone use as the de-pendent variable (Y), social anxiety as the mediating variable (M), age and gender as the control variables, and then subjected to mediating effect analysis using macro program model 4 of the PROCESS software (Table 3, Figure 1). The results showed that physical activity negatively predicted problematic smartphone use (β = −0.07, *P* < 0.001). After social anxiety was included, the negative predictive effect of physical activity on problematic smartphone use was still significant (β = −0.03, *P* = 0.002), while social anxiety positively predicted problematic smartphone use (β = 0.69, *P* < 0.001).

**Table 3:** Tests of mediating effect of social anxiety.

**Model**	**Dependent variable**	**Independent variable**	**β**	***SE***	***t***	***P***	**95%*CI***
Model 1	Mobile phone addiction	Age	0.10	0.24	0.43	0.667	−0.37~0.58
		Gender	1.46	0.48	3.02	0.003	0.51~2.40
		Physical activity	−0.07	0.01	−5.55	< 0.001	−0.09~−0.04
Model 2	Social anxiety	Age	0.04	0.18	0.24	0.808	−0.30~0.39
		Gender	2.67	0.35	7.60	< 0.001	1.98~3.36
		Physical activity	−0.05	0.01	−5.67	< 0.001	−0.07~−0.03
Model 3	Mobile phone addiction	Age	0.07	0.21	0.36	0.722	−0.34~0.49
		Gender	−0.39	0.42	−0.93	0.354	−1.22~0.44
		Physical activity	−0.03	0.01	−3.11	0.002	−0.05~−0.01
		Social anxiety	0.69	0.02	31.41	< 0.001	0.65~0.73

The results of deviation-corrected percentile Bootstrap method showed that social anxiety had a partial mediating effect between physical activity and problematic smartphone use among medical students, with an effect value of −0.04 and a 95% confidence interval of (−0.05~-0.02), accounting for 57.14% of the total effect (Table 4).

**Table 4:** Total, direct and mediating effects of physical activity on problematic smartphone use of medical students.

**Pathway**	**Effective value (95%*CI*)**	**Ratio of effective value (%)**
The direct effect of physical activity → problematic smartphone use	−0.03 (−0.05~−0.01)	42.86
The mediating effect of physical activity → social anxiety → problematic smartphone use	−0.04 (−0.05~−0.02)	57.14
The total effect of physical activity → problematic smartphone use	−0.07 (−0.09~−0.04)	100.00

### The regulation of gender on the mediating effect of social anxiety

The macro program model 14 of the PROCESS software was used to examine whether gender moderates the latter half of the mediating effect of social anxiety between physical activity and problematic smartphone use. The results showed that the interaction terms of gender and social anxiety significantly negatively predicted problematic smartphone use among college students (β = −0.30, *P* < 0.001), that means, gender moderates the second half of the mediating effect of social anxiety between physical activity and problematic smartphone use (Table 5).

**Table 5:** Test of the moderating effect of gender.

**Dependent variable**	**Independent variable**	**β**	***SE***	***t***	***P***	**95%*CI***
Mobile phone addiction	Gender	13.33	2.10	6.35	< 0.001	9.21~17.44
	Physical activity	−0.03	0.01	−3.08	0.002	−0.05~−0.01
	Social anxiety	1.18	0.08	15.47	< 0.001	1.03~1.33
	Gender × social anxiety	−0.30	0.04	−6.67	< 0.001	−0.39~−0.21

**Figure 2:**
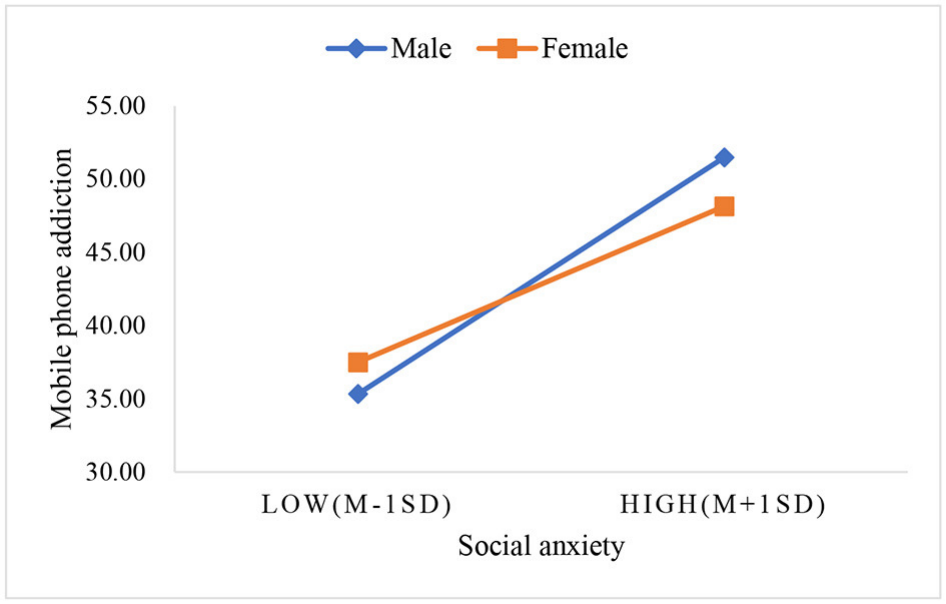
The moderating effect of gender on social anxiety and problematic smartphone use. In the male group, the problematic smartphone use score increases synchronously with the social anxiety score, with a slope of 0.879 (*t* = 24.703, *P* < 0.001). Conversely, in the female group, the problematic smartphone use score also increased with the increase of social anxiety score, the slope was 0.580 (*t* = 21.004, *P* < 0.001), suggesting a slower rate of increase.

To better explain the moderating effect of gender, social anxiety group was divided into high social anxiety group and low social anxiety group according to the mean plus or minus one standard deviation, and a simple slope plot was drawn. As shown in Figure 2, in the male group, the problematic smartphone use score increases synchronously with the social anxiety score, with a slope of 0.879 (t = 24.703, *P* < 0.001), the problematic smartphone use score in female group also in-creased with the increase of social anxiety score, the slope was 0.580 (t = 21.004, *P* < 0.001), but the rate of increase is slower.

## Discussion

Problematic smartphone use has emerged as a significant global public health issue (Tu et al., 2023). Early identification, intervention, and improvement of health literacy among medical students are key research priorities in the academic community (Jahagirdar et al., 2021). This study targets medical students to investigate the relationship between physical activity and problematic smartphone use, as well as the mediating role of social anxiety. The findings suggest that physical activity can directly and indirectly affect problematic smartphone use through social anxiety among college students. Moreover, gender plays a moderating role in the interaction between social anxiety and problematic smartphone use, highlighting the importance of considering gender differences in addressing problematic smartphone use among this population. It reveals the internal mechanism through which physical activity on problematic smartphone use, offering insights for guiding the intervention of the problematic smartphone use.

This study confirms that physical activity is negatively correlated with problematic smartphone use, supporting the notion that increase of physical activity level can reduce the tendency of problematic smartphone use, which is consistent with previous research results (Xiao et al., 2021; Tong et al., 2022). Hypothesis (1) has thus been verified. According to the self-control resource model theory, the self-control resources of each person are limited, and the self-control behavior will consume certain resources (Inzlicht and Schmeichel, 2012; Xian and Hua, 2019). Regular physical activity can replenish the resources of self-control, improve the ability of self-control and reduce the tendency of problematic smartphone use. In addition, physical activity stimulates the pituitary gland to release endorphins, enhance the ability of human dopamine signal transduction, promote the generation of positive emotions, and effectively alleviate the discomfort of medical students when they disconnect from mobile phones (Zhang, 2012). By actively engaging in diverse sports activities and fostering healthy habits, individuals can reduce both the duration and frequency of mobile phone use, thereby mitigating problematic smartphone use.

The additional analysis in this study indicates that social anxiety serves as a partial mediator between physical activity and problematic smartphone use, thereby confirming hypothesis (2). Specifically, physical activity is shown to negatively influence social anxiety, while social anxiety positively predicts problematic smartphone use. The cognitive-behavioral theory of Davis suggests that social anxiety is a critical element of addictive behavioral psychosis (Zhu, 2017). Individuals with a high levels of social anxiety may overly focus on self-perception, lack confidence in their appearance, speech and behavior, and fear being scrutinized by others in interpersonal communication (Meleshko and Alden, 1993). Mobile phones, offering secretive and virtual, environment, allow these individuals seek comfort from strangers online, build a positive self-image, display personal abilities, and obtain a better sense of social control to meet their social needs, thus exacerbating tendencies toward problematic smartphone use (Liao et al., 2017; Zhu, 2017). Participation in group sports such as football, basketball, relay competition, enhances social interaction group cooperation and skill development, reduces fear of social engagement, and gradually diminishes reliance on mobile phones for social fulfillment (Tong et al., 2022). These activities provide practical avenues for overcoming social anxiety and its contribution to problematic smartphone use.

This study also found that gender plays a moderating role in the relationship between social anxiety and mobile phone addiction among medical students, i.e., the role is different between male and female groups. The finding is contrary to hypothesis (3), which is specifically manifested in the following: the male group tends to use mobile phones more frequently than the female group to alleviate social anxiety problems after they occur. This may be because when faced with social distress, females are more likely to seek social support from people around them, reassess the severity of negative events and establish positive evaluations to relieve anxiety (McRae et al., 2008; Nolen-Hoeksema, 2012). Conversely, males have more positive attitudes toward electronic products, games, virtual socializing, etc., and are more likely to indulge in high-risk online behaviors, such as gambling, browsing pornographic websites, etc. (Tsitsika et al., 2009). This conclusion shows that social anxiety is an important factor influencing mobile phone addiction among male groups. Therefore, in the research and educational work, the differences between different genders should be fully considered, more attention should be paid to the spiritual life of the male group, the management and supervision of their online activities should be strengthened, and they should be helped to stay away from high-risk online behaviors, to establish positive cognitive assessment of social anxiety, and to prevent the mobile phone addiction problem.

Mobile phone addiction is a major hidden danger to the mental health of contemporary college students. Due to their special disciplinary background, medical students are more likely to fall into mobile phone addiction when faced with more severe academic and social pressure. College administrators should recognize the seriousness of the problem of mobile phone addiction among medical students, encourage students to assess the risk of mobile phone addiction through online tools, and become aware of this problem for early identification and appropriate intervention; formulate health education plans and actively organize various sports activities, guide medical students to use their spare time to cultivate a healthy lifestyle, so as to better cope with academic and social pressures, alleviate mobile phone addiction problems, and improve the professionalism of medical students in future medical careers.

### Research limitation

This study has several limitations due to its scope and design that need to be addressed in the future research. Firstly, the research subjects are selected from freshmen and sophomores from a medical college in various regions of Shandong, China. The specific and relative small demographic may not provide a comprehensive understanding the causal relationship between physical exercise and mobile phone addiction, thus limiting the generalizability of the findings. Future studies should expand the sample size to include a broader demographic across all levels of medical education and conduct longitudinal studies to provide stronger evidence of the correlations the correlation between physical activity, mobile phone addiction and social anxiety. Secondly, the cross-sectional design of this study restricts its ability to reveal the causal relationship between variables, and future attempts should be made to carry out longitudinal and experimental research in order to test the results of the current study. Finally, while our study identified the mediating effect of social anxiety and the moderating role of gender, other factors may also influence the dynamic. Future research could further examine additional mechanism and factors that impact the relationship between physical activity and mobile phone addiction.

## Conclusion

The study establishes a negative correlation between physical exercise and problematic smartphone use among medical students. Physical exercise impacts problematic smartphone use both directly and indirectly through its influence on social anxiety. Additionally, gender significantly moderates the relationship between social anxiety and problematic smartphone use. These findings provide important insights into the underlying mechanisms of how physical exercise influences problematic smartphone use and underscore the importance of incorporating physical activity into interventions and prevention strategies for problematic smartphone use among medical students.

## Data Availability

The original contributions presented in the study are included in the article/supplementary material, further inquiries can be directed to the corresponding author.
